# First Behavioural Characterisation of a Knockout Mouse Model for the Transforming Growth Factor (TGF)-β Superfamily Cytokine, *MIC-1/GDF15*

**DOI:** 10.1371/journal.pone.0168416

**Published:** 2017-01-12

**Authors:** Jac Kee Low, Ananthan Ambikairajah, Kani Shang, David A. Brown, Vicky W. W. Tsai, Samuel N. Breit, Tim Karl

**Affiliations:** 1 Neuroscience Research Australia (NeuRA), Randwick, New South Wales, Australia; 2 School of Medicine, Western Sydney University, Campbelltown, New South Wales, Australia; 3 St Vincent’s Centre for Applied Medical Research, St Vincent’s Hospital, Darlinghurst, New South Wales, Australia; 4 Westmead Institute for Medical Research, The Institute for Clinical Pathology and Medical Research and Westmead Hospital, Westmead, New South Wales, Australia; Hokkaido Daigaku, JAPAN

## Abstract

Macrophage inhibitory cytokine-1 (MIC-1), also known as growth differentiation factor 15 (GDF15), is a stress response cytokine. MIC-1/GDF15 is secreted into the cerebrospinal fluid and increased levels of MIC-1/GDF15 are associated with a variety of diseases including cognitive decline. Furthermore, *Mic-1/Gdf15* knockout mice (*Mic-1* KO) weigh more, have increased adiposity, associated with increased spontaneous food intake, and exhibit reduced basal energy expenditure and physical activity. The current study was designed to comprehensively determine the role of *MIC-1/GDF15* on behavioural domains of male and female knockout mice including locomotion, exploration, anxiety, cognition, social behaviours, and sensorimotor gating. *Mic-1* KO mice exhibited a task-dependent increase in locomotion and exploration and reduced anxiety-related behaviours across tests. Spatial working memory and social behaviours were not affected by *Mic-1*/*Gdf15* deficiency. Interestingly, knockout mice formed an increased association with the conditioned stimulus in fear conditioning testing and also displayed significantly improved prepulse inhibition. Overall sex effects were evident for social behaviours, fear conditioning, and sensorimotor gating. This is the first study defining the role of *Mic-1*/*Gdf15* in a number of behavioural domains. Whether the observed impact is based on direct actions of *Mic-1*/*Gdf15* deficiency on the CNS or whether the behavioural effects are mediated by indirect actions on e.g. other neurotransmitter systems must be clarified in future studies.

## 1. Introduction

Macrophage inhibitory cytokine-1 (MIC-1), also known as growth differentiation factor 15 (GDF15), is a stress response cytokine that is a divergent member of the transforming growth factor (TGF)-β superfamily. The *MIC-1/GDF15* gene is on chromosome 19p12-13.1 and consists of two exons separated by an intronic sequence of about 1800 bp [1]. Under normal physiological conditions, the placenta is the only tissue that expresses large amounts of MIC-1/GDF15 [2]. However, it is expressed in low amounts in the liver, lung and kidney as well as adipocytes and a variety of epithelial cells, including the choroid plexus [2–4]. MIC-1/GDF15 is present in the serum of all individuals with a normal range of 150–1150 pg/ml, but these serum levels increase further with injury, inflammation and malignancy. Modestly elevated serum levels are associated with and predict the outcome of a wide variety of disease processes including cardiovascular diseases, diabetes, cognitive decline and cancer. MIC-1/GDF15 blood levels are also a powerful predictor of all-cause mortality, suggesting a fundamental role in biological processes associated with ageing [5]. In some disease states such as chronic renal and cardiac failure and advanced cancer, MIC-1/GDF15 expression is dramatically increased by up to 10–100 fold [3, 6–8].

When MIC-1/GDF15 is markedly overproduced, such as in advanced cancer, it leads to an anorexia/cachexia syndrome by acting on brain feeding centers in the hypothalamus and hindbrain. Mice transgenically overexpressing Mic-1/Gdf15 have a lean phenotype and resist obesity [9]. Conversely, *Mic-1/Gdf15* gene knockout mice weigh more and have increased adiposity, which is associated with increased spontaneous food intake. Female knockout mice also exhibit reduced basal energy expenditure and physical activity, possibly owing to the associated decrease in total lean mass [10]. Thus, *Mic-1/Gdf15* is involved in the physiological regulation of appetite and energy storage, a process that becomes subverted in some disease states leading to anorexia/cachexia.

Pleiotropic actions have been described for human *MIC-1/GDF15*. Furthermore, MIC-1/GDF15 is secreted into the cerebrospinal fluid [11] and is able to act on at least some brain centres as well as having neurotrophic and possibly neuroprotective effects. Thus, the current study was designed to comprehensively determine for the first time the role of *MIC-1/GDF15* in laboratory mouse behaviour beyond food intake and physical activity. For this, adult control and germline *Mic-1/Gdf15* knockout mice were characterised in behavioural paradigms relevant to locomotion, exploration, anxiety, cognition, social behaviours, and sensorimotor gating. Importantly, sex-dependent differences were also considered, which is in line with good scientific practice when evaluating a newly developed mouse model. This strategy also follows up on earlier findings of sex-specific food intake and metabolic phenotypes of these mice [10].

## 2. Materials and methods

### 2.1 Animals

Mice with germline-deleted *Mic-1/Gdf15* (from now on: *Mic*-1 KO or *Mic-1*^-/-^) were generated by Ozgene (Ozgene Pty Ltd., Bentley DC, Australia). These mice have a complete deletion of the second of two exons of the *Mic-1*/*Gdf15* gene. This effectively deleted the poly-A tract and amino acids 94–302 of Mic-1/*Gdf15*, including all of the mature bioactive domain and most of the pro-peptide region. The founder mice were bred for more than 10 generations onto a C57BL/6JAbr background. *Mic-1* KO mice (*n* = 12-13/sex) and wild type-like control littermates (WT: *n* = 12-13/sex) were bred and group-housed in independently ventilated cages (Airlaw, Smithfield, Australia) at Animal BioResources (Moss Vale, Australia). Test mice of both sexes were transported to Neuroscience Research Australia (NeuRA) at around 10 weeks of age (±1 week), where they were group-housed in Polysulfone cages (1144B: Techniplast, Rydalmere, Australia) equipped with some tissues for nesting. Mice were kept under a 12: 12 h light: dark schedule [light phase: white light (illumination: 124 lx)–dark phase: red light (illumination: < 2 lx)]. Food and water were provided *ad libitum*, except where specified. Adult, sex-matched A/J mice from Animal Resources Centre (Canning Vale, Australia) were used as standard opponent for the social interaction test. Research and animal care procedures were approved by the University of New South Wales Animal Care and Ethics Committee in accordance with the Australian Code of Practice for the Care and Use of Animals for Scientific Purposes.

### 2.2 Behavioural phenotyping

Starting at 5 months of age (±1 week), mice were tested in a battery of behavioural tests with an inter-test interval of at least 48 h. All tests were conducted during the first 5 h of the light phase to minimise effects of the circadian rhythm on the performance of test mice. The test order was as follows: elevated plus maze, open field, spontaneous alternation, social interaction, prepulse inhibition, fear conditioning, and physical exam.

#### 2.2.1 Elevated plus maze (EPM)

The EPM assesses the natural conflict between the tendency of mice to explore a novel environment and avoidance of a brightly lit, elevated and open area [12]. The grey plus maze was “+” shaped (for details of apparatus see [13]). Mice were placed at the centre of the + (faced towards an enclosed arm) and were allowed to explore the maze for 5 min. Locomotion was recorded both as distance travelled and as arm entries. Furthermore, the percentage time spent and percentage distance travelled in the open arms (as a measure of total arm performance) were recorded as anxiety measures using Any-Maze^™^ (Stoelting, Wood Dale, USA) tracking software.

#### 2.2.2 Open field (OF)

The OF mimics the natural conflict in mice between the tendency to explore a novel environment and to avoid an exposed open area [14, 15]. Mice were placed into an infrared photobeam controlled open field activity test chamber (MED Associates Inc., USA, Vermont). The arena (43.2 cm x 43.2 cm) was divided into a central and a peripheral zone (MED Associates Inc software coordinates for central zone: 3/3, 3/13, 13/3, 13/13). The animal’s horizontal activity (i.e. distance travelled), vertical activity (i.e. *rearing*), small motor movements (i.e. movements below the ambulation threshold), and *resting* behaviour (no infrared photobeam-detectable movements), were recorded automatically for the different zones (software settings for ambulation threshold: box size: 3; ambulatory trigger: 2; resting delay: 1000 ms; resolution: 100 ms). The ratio of central to total distance travelled and time spent in the central zone were taken as measures of anxiety [16].

#### 2.2.3 Continuous spontaneous alternation in the Y-maze (SA)

The Y-maze SA test measures the willingness of mice to explore novel environments. Rodents typically prefer to investigate a new arm of a maze rather than returning to one that was previously visited [17]. The Y-maze used in our laboratory consisted of three grey acrylic arms (10 cm x 30 cm x 17 cm) placed at 120° with respect to each other. Around the arms were distal cues. Animals were placed into the centre of the Y-maze and allowed to freely explore the environment for 10 min. Order of entries into the three different arms (A, B, or C) was recorded and successful arm entry triplets (i.e. ABC, ACB, BCA, BAC, CAB, CBA) calculated (maximal number of correct triplets = total number of arm entries– 2). An arm entry was scored whenever an animal entered an arm with more than half of its body length.

#### 2.2.4 Social interaction test (SI)

The SI paradigm is used to measure social behaviours. Test mice and sex and age-matched A/JArc standard opponents were placed in opposite corners of a grey PVC arena (300 mm x 350 mm x 350 mm) and were allowed to explore the arena and each other freely for 10 min. The frequency and duration of the following active socio-positive behaviours were recorded: *general sniffing*, *anogenital sniffing*, *following* and *climbing over* [18, 19]. Total distance travelled was measured as a general activity score using Any-Maze^™^.

#### 2.2.5 Fear Conditioning (FC)

Fear conditioning assesses associative learning whereby a previously neutral stimulus elicits a fear response after it has been paired with an aversive stimulus. On conditioning day, mice were placed into the test chamber (Model H10-11R-TC: Coulbourn Instruments, USA) for 2 min. Then an 80 dB conditioned stimulus (CS) was presented for 30 seconds with a co-terminating 0.4 mA 2 second foot shock (unconditioned stimulus; US) twice with an inter-pairing interval of 2 min). The test concluded 2 min later. The next day (context test), mice were returned to the apparatus for 7 min. On day 3 (cue test), animals were placed in an altered context for 9 min. After 2 min (pre-CS/baseline), the CS was presented continuously for 5 min. The test concluded after another 2 min with the absence of the CS. Time spent *freezing* was measured using Any-Maze^™^ software [20, 21]. Three mice (1x male *Mic*-1 KO, 1x female WT, and 1x male WT) jumped out of the test chamber during conditioning and were therefore not included in any further FC testing or the statistical analysis for this paradigm.

#### 2.2.6 Sensorimotor gating (i.e prepulse inhibition: PPI)

PPI, an operational measure of sensorimotor gating, is the attenuation of the startle response by a non-startling stimulus (prepulse) presented before the startling stimulus (pulse). Test mice were placed in Plexiglas mouse enclosures of the startle chambers (SR-Lab: San Diego Instruments, San Diego, USA) with a 70 dB consistent background noise and allowed to habituate to the enclosure and test apparatus for 5 min over 3 consecutive days prior to PPI testing. The 30 min PPI test session consisted of a 5 min acclimation period to 70dB background noise, followed by 97 trials presented in a pseudorandom order: 5 x 70dB trials; 5 x 100dB trials; 15 x 120dB trials to measure the acoustic startle response (ASR) and 15 sets of 5 trials comprising of a prepulse of either 74, 82 or 86dB presented 32, 64, 128, or 256 ms (variable interstimulus interval; ISI) prior to a startle pulse of 120dB to measure the PPI response. The intertrial interval (ITI) varied randomly from 10–20 seconds. Responses to each trial were calculated as the average mean amplitude detected by the accelerometer [22, 23].

For ASR analysis, mean ASR was assessed for different startle pulses (i.e. 70/100/120dB), for ASR habituation analysis, blocks of ASR to 120dB were averaged at the beginning, middle and end of the PPI protocol (5 trials per block) and compared. The overall ASR was calculated as the mean amplitude to middle startle trials (as habituation effects were detected, see Results) and percentage PPI (%PPI) was calculated as [(middle startle block response (120dB)–PPI response)/middle startle block response (120dB)] x 100. %PPI was averaged across ISIs to produce a mean %PPI for each prepulse intensity.

### 2.3 Statistical analysis

Analysis of the behavioural parameters was performed using two-way analysis of variance (ANOVA) to investigate main effects and interactions between ‘genotype’ and ‘sex’ or repeated measures (RM) ANOVAs for effects of ‘5 min block‘ (OF), ‘1 min block’ (FC), ‘startle pulse’, ‘startle block’ and ‘prepulse intensity’ (all PPI) as published previously [13]. In line with Rothman and Perneger the data were not adjusted for multiple comparisons and were interpreted as such in the discussion [24, 25]. Differences were regarded as significant if *p* < .05. F-values and degrees of freedom are presented for ANOVAs. Data are shown as means ± standard error of means (SEM). Analyses were conducted using Statview software Version 5.0.

## 3. Results

All processed data are shown in S1 File.

### 3.1 Locomotion and exploration

**Fig 1:**
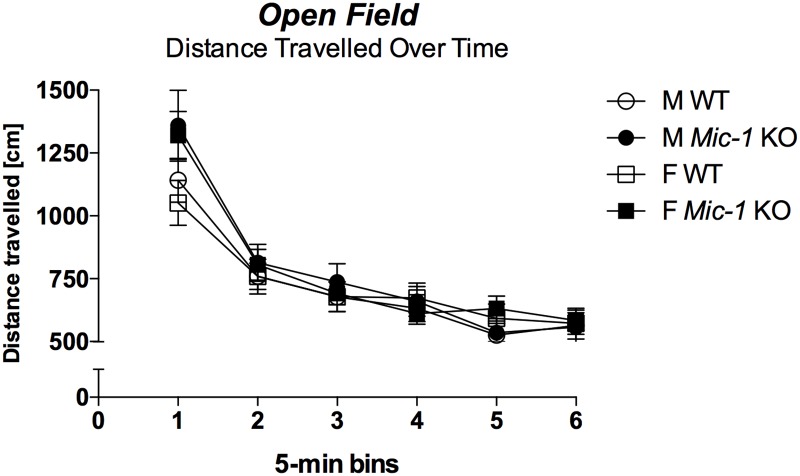
Habituation of locomotive response to novelty in the open field (OF). Overall distance travelled in the open field [cm] across 5-min blocks. Data for control (WT) and *Mic-1* knockout mice (*Mic-1* KO) are shown for males (M) and females (F) as means + SEM. There was a significant ‘5 min block’ x ‘genotype’ interaction (*p* < .0009).

2way ANOVA revealed that *Mic-1* KO mice exhibit a subtle, task-specific increase in locomotor activity compared to control mice, but only in the EPM for number of arm entries [F(1,48) = 7.4, *p* = .009] but not the distance travelled in all arms [F(1,48) = .5, *p* = .5] or the total distance travelled in the OF test [F(1,48) = 1.2, *p* = .3] (Table 1). However, the *Mic-1* genotype impacted significantly on the habituation of the locomotive response to a novel environment in the latter test with *Mic-1* KO mice displaying a stronger reduction of the locomotor response over time (i.e. distance travelled in the OF across 5 min blocks) compared to control animals [RM ANOVA ‘5 min block’ x ‘genotype’: F(5,240) = 4.3, *p* < .0009] (Fig 1). A similar interaction was found for peripheral distance travelled over time (data not shown). There were no interactions between ‘genotype’ and ‘sex’ for any of the parameters investigated. *Mic-1* KO mice also showed increased vertical activity (i.e. *rearing*) [trend only; F(1,48) = 3.1, *p* = .08] and small motor movements [F(1,48) = 11.7, *p* = .001] across sex in the OF (Table 1).

**Table 1:** Behaviours of wild type-like control (WT) and *Mic-1* knockout mice (*Mic-1* KO) of both sexes in elevated plus maze (EPM), the open field (OF), the spontaneous alternation task (SA), the social interaction test (SI), and prepulse inhibition (PPI). There was a main effect of ‘genotype’ for number of arm entries in the EPM [F(1,48) = 7.4, *p* = .009] and for small motor movements [F(1,48) = 11.7, *p* = .001] as well as for percentage prepulse inhibition [%PPI: F(1,48) = 11.5, *p* = .001].

	WT	*Mic-1* KO	WT	*Mic-1* KO
	Male	Male	Female	Female
EPM—Arm entries [n]	22.5 ± 2.1	29.3 ± 2.5	24.8 ± 1.2	30.0 ± 2.8
EPM—Distance travelled [cm]	1049.1 ± 73.7	1161.7 ± 83.3	1143.1 ± 61.0	1209.5 ± 115.2
OF—Distance travelled [cm]	4301.0 ± 302.8	4661.9 ± 370.6	4327.5 ± 313.3	4644.3 ± 270.4
OF—Vertical activity [n]	232.9 ± 18.9	289.7 ± 24.3	211.9 ± 31.4	242.2 ± 22.2
OF—Small motor movements [n]	2318.5 ± 36.3	2388.1 ± 52.1	2152.3 ± 61.8	2228.9 ± 35.0
SA—Arm entries [n]	27.9 ± 2.5	31.3 ± 3.3	31.5 ± 3.2	32.0 ± 2.2
SA—Spontaneous alternation [%]	49.6 ± 3.0	54.8 ± 3.4	52.3 ± 3.8	53.8 ± 2.7
SI—Social interaction time [s]	68.5 ± 4.5	58.9 ± 4.6	72.8 ± 4.1	76.0 ± 7.0
PPI—Average percentage PPI [%]	41.8 ± 3.7	56.9 ± 4.1	32.8 ± 4.3	50.6 ± 4.0

### 3.2 Anxiety

**Fig 2:**
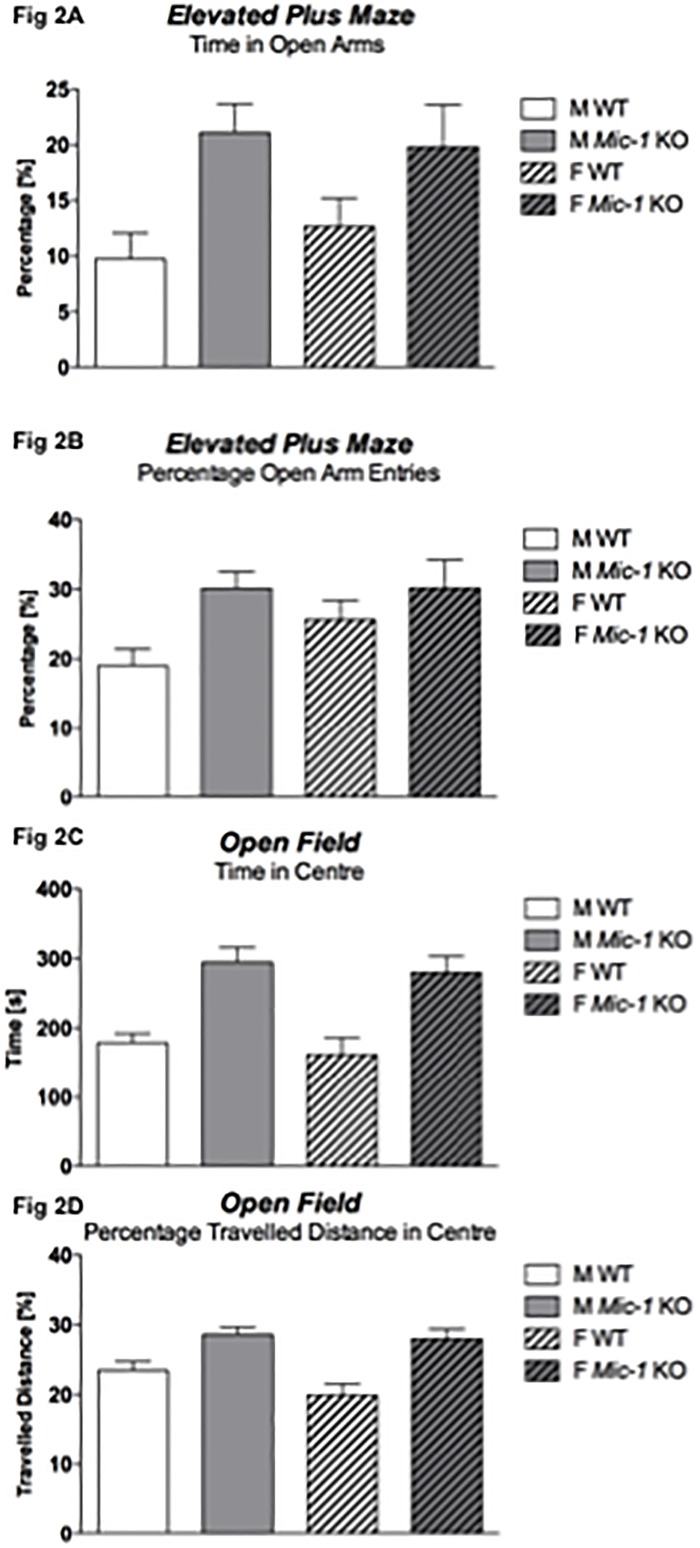
Anxiety-related behaviours in the elevated plus maze (EPM) and the open field test (OF). (A) Percentage time spent on open arms (excluding centre time) [%], (B) percentage of entries into open arms [%], (C) time spent in the central zone of the OF [s], and (D) ratio of total distance travelled in the central zone of the OF. Data for control (WT) and *Mic-1* knockout mice (*Mic-1* KO) of both males (M) and females (F) are shown as means + SEM. In the EPM, *Mic-1* mice spent more time (*p* = .002) and also showed more locomotion (*p* = .01) in the open arms. In the OF, knockout mice spent more time (*p* < .0001) and showed more locomotion (*p* < .0001) in the centre.

*Mic-1* deficient mice displayed significantly less anxiety-related behaviours in both EPM and OF compared to WT littermates, indicating a very robust anxiolytic-like phenotype. In the EPM, *Mic-1* KO mice of both sexes spent more time in the more aversive open arms [percentage open arm time: F(1,48) = 10.4, *p* = .002; Fig 2A] and also showed more locomotion in those arms [percentage open arm entry: F(1,48) = 7.0, *p* = .01; Fig 2B]. In support of these findings, time spent and percentage distance travelled in the centre of the OF were significantly increased in knockout mice as well [time: F(1,48) = 31.1, *p* < .0001 –ratio: F(1,48) = 22.6, *p* < .0001] and (Fig 2C and 2D). No interactions between ‘genotype’ and ‘sex’ were detected.

### 3.3 Cognition

There were no effects of ‘genotype’ or ‘sex’ on the locomotive phenotype (i.e. arm entries) in the Y-Maze. Furthermore, there were no main effects on the cognitive behaviour (i.e. percentage of spontaneous alternation) investigated in the spontaneous alternation task (Table 1).

**Fig 3:**
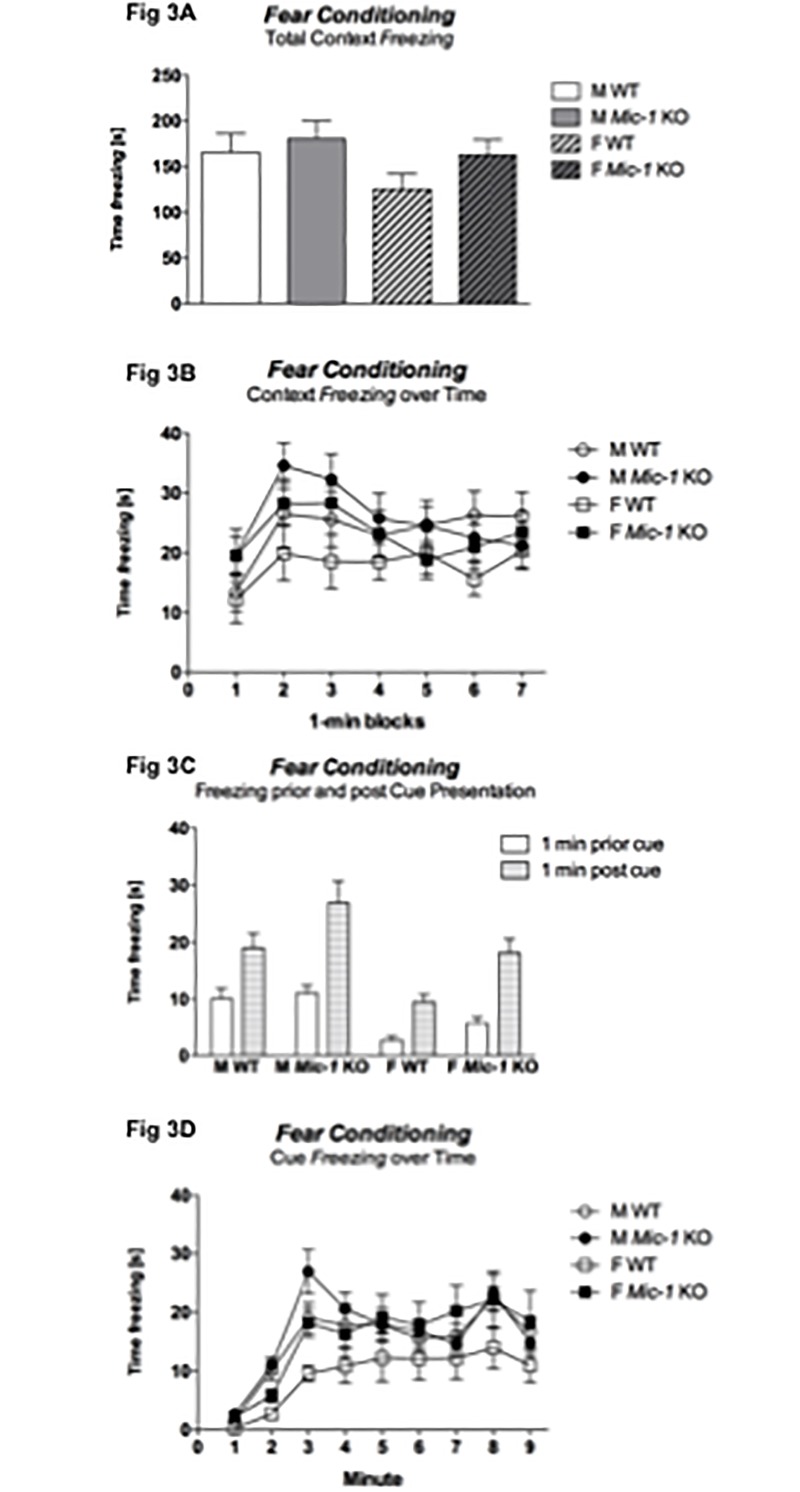
Fear-associated memory to context and cue. (A) Total time spent *freezing* [s] during the context test, (B) time spent *freezing* [s] across 1-min blocks in the context test, (C) time spent *freezing* [s] in the 1-min block prior and post cue presentation, and (D) time spent *freezing* [s] across 1-min blocks during cue presentation. Data for control (WT) and *Mic-1* knockout mice (*Mic-1* KO) of both males (M) and females (F) are shown as means + SEM. Comparing freezing in the last min prior to cue onset with the first min post cue onset revealed effects of ‘sex’ (*p* < .0001) and ‘genotype’ (*p* = .005) as well as a significant ‘1 min block’ by ‘genotype’ interaction (*p* = .01). Three mice (1x male *Mic*-1 KO, 1x female WT, and 1x male WT) jumped out of the test chamber during conditioning and were therefore not included in the statistical analysis.

In the context version of the FC, neither sex nor genotype significantly impacted on the total *freezing* response to the context [‘sex’: F(1,45) = 2.4, *p* = .1 - ‘genotype’: F(1,45) = 2.0, *p* = .2] (Fig 3A). However, there was a trend for ‘1 min block’ by ‘genotype’ interaction for context freezing across time F(6,270) = 1.9, *p* = .09] with *Mic-1* KO mice showing an increased *freezing* response across time (Fig 3B).

In the cue version of this task, comparing the last minute before cue onset with the first minute post cue onset revealed overall effects of ‘sex’ [F(1,45) = 19.9, *p* < .0001] and ‘genotype’ [F(1,45) = 8.7, *p* = .005] as well as a significant ‘1 min block’ by ‘genotype’ interaction [F(1,45) = 7.0, *p* = .01] with males and *Mic-1* KO mice showing increased *freezing* responses to the cue compared to the corresponding animals (i.e. females and WT mice respectively: Fig 3C). Nonetheless, split for ‘genotype’ revealed that both WT and *Mic-1* KO mice exhibited increased *freezing* post cue onset (data not shown). There was also a trend for a ‘genotype’ effect for *freezing* across the full period of cue presentation [F(1,45) = 3.0, *p* = .09; Fig 3D].

### 3.4 Social interaction

There was a main effect of ‘sex’ on the duration of active social behaviours [F(1,48) = 4.3, *p* < .05], with female mice showing more active social interaction than male counterparts (Table 1). There were no significant main effects of ‘genotype’ on active social interaction time or individual social behaviours [except trends for frequencies of *crawling under* (*p* = .07) and *allo-grooming* (*p* = .06)] and no ‘genotype’ by ‘sex’ interactions (data not shown).

### 3.5 Acoustic startle response and sensorimotor gating

**Fig 4:**
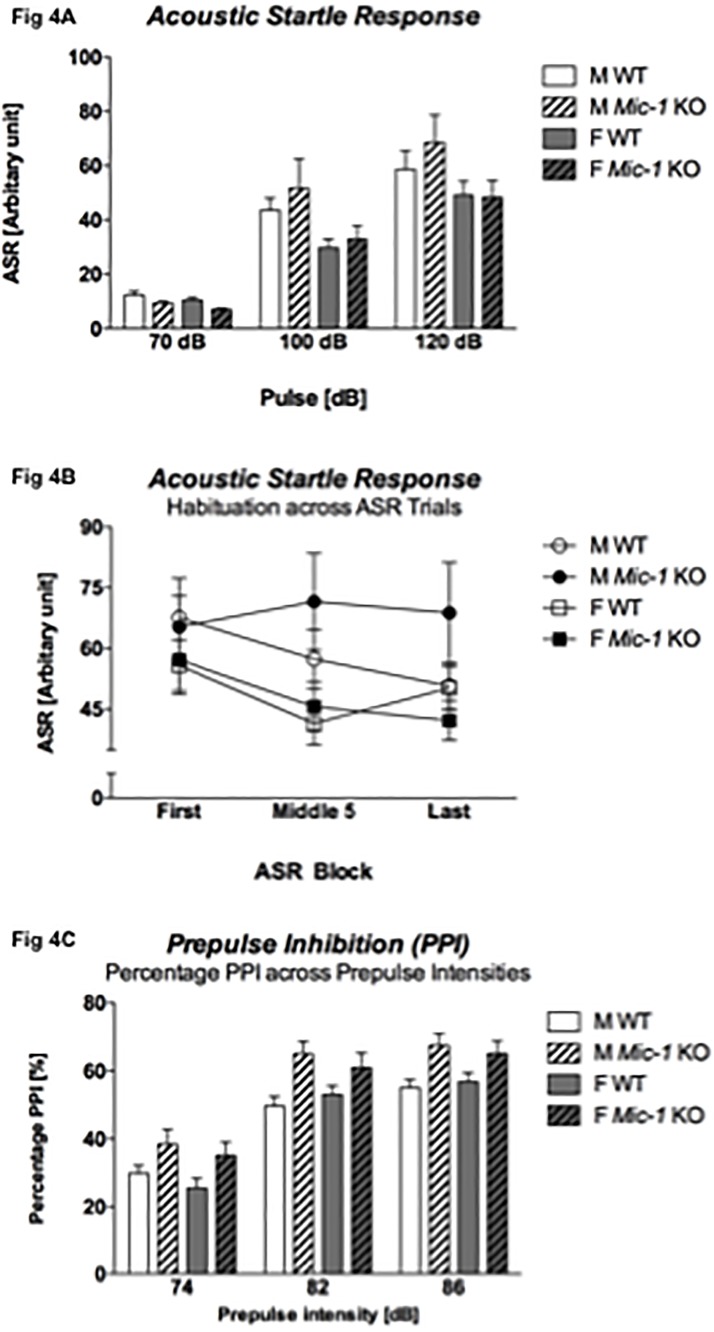
Acoustic startle response (ASR) and sensorimotor gating (i.e. prepulse inhibition: PPI). (A) ASR to different acoustic startle stimuli, (B) habituation to a 120dB startle stimulus across test trials (i.e. averaged across 3 blocks of 5 trials each), and (C) percentage prepulse inhibition [%PPI] across different prepulse intensities (i.e. 74dB, 82dB, and 86dB)–calculated for the middle 120dB ASR block. Data for control (WT) and *Mic-1* knockout mice (*Mic-1* KO) of both males (M) and females (F) are shown as means + SEM. A significant ‘sex’ effect (*p* < .05) and ‘startle block’ by ‘sex’ by ‘genotype’ interaction effect (*p* = .03) were found for ASR habituation. Only female mice and control mice (both *p* = .002) displayed intact ASR habituation. %PPI across prepulse intensities revealed a main effect of ‘genotype’ (*p* < .001) and a ‘sex’ by ‘prepulse intensity’ interaction (*p* = .01).

The genotype had no impact on the acoustic startle response to various startle pulses [RM ANOVA, ‘genotype’ by ‘startle’: F(2,96) = 1.2, p = .3] but there was a strong trend for a ‘sex’ by ‘startle’ interaction [F(2,96) = 3.0, *p* = .06] (Fig 4A). Furthermore, habituation to a 120dB startle stimulus across trials revealed both a main effect of ‘sex’ [F(1,48) = 4.1, *p* < .05] and a significant ‘startle block’ by ‘sex’ by ‘genotype’ interaction [RM ANOVA: F(2,96) = 3.6, *p* = .03]. Split by ‘sex’, only female mice habituated to a 120dB stimulus [RM ANOVA for females: F(2,48) = 10.3, *p* = .002 –males: not significant] (Fig 4B). Split by ‘genotype’, only control WT mice displayed ASR habituation [RM ANOVA for WT: F(2,48) = 6.9, *p* = .002 –*Mic-1* KO: not significant] (Fig 4B). Thus, for the following analysis, PPI was calculated as a percentage of the middle ASR startle block rather than of the ASR averaged across all three startle blocks.

Analysis of PPI to increasing prepulse intensities revealed a significant effect of ‘prepulse intensity’ on %PPI across mice [RM ANOVA: F(2,96) = 306.8, *p* < .001]. There was a main effect of ‘genotype’ [F(1,48) = 16.3, *p* < .001] with *Mic-1* KO mice displaying significantly higher %PPI [no ‘genotype’ by ‘prepulse intensity’ interaction: F(2,96) = .4, *p* = .7] (Fig 4C). There was also a trend for a ‘genotype’ by ‘sex’ by ‘prepulse intensity’ interaction [F(2,96) = 2.7, *p* = .07] and more importantly, a significant ‘sex’ by ‘prepulse intensity’ interaction [F(2,96) = 4.8, *p* = .01] with female mice showing more pronounced %PPI with increasing prepulse intensities (Fig 4C). The average prepulse inhibition response was affected by ‘genotype’ as well [F(1,48) = 16.3, *p* < .001] with *Mic-1 KO* mice displaying more robust PPI than their control littermates regardless of sex (Table 1).

## 4. Discussion

Here we present the very first report on the consequences of germline depletion of *Mic-1*/*Gdf15* on various behaviours of laboratory mice. Male and female adult mice were characterised in behavioural paradigms with relevance to locomotion/exploration, anxiety, cognition, social behaviours and sensorimotor gating. *Mic-1* KO mice exhibited a moderate increase in locomotion in the EPM and in exploration in the OF. Furthermore, the locomotor response to a novel environment habituated faster in these mice compared to control animals. In line with this, *Mic-1*/*Gdf15* deficient mice regardless of sex displayed significantly less anxiety-related behaviours across tests. Spatial working memory as evaluated using the YM and social behaviours were not affected by *Mic-1*/*Gdf15* deficiency. Interestingly, knockout mice displayed an increased association with the CS in the cue version of the fear conditioning test. *Mic-1* KO mice also displayed significantly improved prepulse inhibition compared to their control littermates. Finally, the sex of the test animals modulated social behaviours, fear conditioning, and sensorimotor gating across genotypes.

So far, the role of *MIC-1*/*GDF15* in brain-related processes has not been assessed in detail and its impact on behavioural domains beyond feeding behaviour has not been studied. However, there is evidence to indicate that it has at least some direct actions on the central nervous system. Interestingly, the effects of systemic Mic-1/Gdf15 administration and genetic overexpression in laboratory mice, hypophagia and body weight, were linked to hypothalamic reduction of neuropeptide Y (NPY) and growth hormone-releasing hormone (GHRH) mRNA as well as up-regulation of pro-opiomelanocortin (POMC). *In situ* hybridization confirmed that NPY and POMC neurons were major targets for MIC-1/GDF15 [8].

Considering that *Mic-1*/*Gdf15* deficiency might result in up-regulation of NPY, it is interesting to note that NPY overexpressing mice show similar behavioural characteristics to *Mic-1* KO mice including decreased anxiety [26]. In line with this, NPY deficient mice demonstrate the ‘opposite’ phenotype with suppressed levels of locomotion and exploration and a pronounced anxiogenic-like response across sex [13]. Finally, the anxiolytic-like action of excess NPY has been confirmed in pharmacological rodent models testing exogenously administered NPY in a variety of anxiety paradigms [27–29]. Interestingly, a germline knockout model for the main NPY receptor subtype Y_1_ has been found to show unaltered sensorimotor gating compared to control mice [30], whereas our *Mic-1* KO mice exhibited a more robust prepulse inhibition phenotype.

Another potential mechanism for the behavioural phenotype of *Mic-1* KO mice is related to the actions of MIC-1/GDF15 on POMC neurons. POMC is a precursor protein, which undergoes post-translational cleavage into several peptides including adrenocorticotropic hormone (ACTH). ACTH regulates the secretion of glucocorticoids from the adrenal cortex thereby impacting on stress/anxiety responses [31]. *Mic-1* KO mice might be expected to have reduced POMC expression, which could then result in reduced glucocorticoid secretion and the anti-anxiety phenotype observed in our *Mic-1* KO mouse model.

It has been suggested that MIC-1/GDF15 may play a role in the induction of interleukin 6 (IL-6)-related inflammatory responses *in vitro* and *in vivo* [32] and its levels are weakly correlated to IL-6 in humans (r = 0.35; [33]). IL-6 is known to have central actions, is elevated in the elderly, and is associated with cognitive impairments and decline (reviewed in [34]). Il-6-deficient animals show higher locomotor activity in an open field and lower levels of exploration of the open arms of the elevated plus maze than control animals [35]. This is in line with what we found in *Mic-1* deficient mice, which may have reduced IL-6 signalling as a direct or indirect consequence of *Mic-1* gene deletion.

Finally, elevated levels of MIC-1/GDF15 appear associated with lower global cognitive performance (e.g. executive functioning and memory) in the elderly, even after correction for e.g. IL-6 and apolipoprotein ε4 genotype [34]. On the contrary, reduced levels of MIC-1/GDF15 might have positive effects on cognition, as our *Mic-1* deficient mice exhibited improved fear-associated memory and sensorimotor gating. A recent review on the role of MIC-1/GDF15 in cognitive ageing and dementia concluded that this TGF-β family member should be considered as a marker for age-related cognitive decline and structural brain changes [36]. The pathophysiology of the relationship is not well understood although it is rather unlikely that elevated MIC-1/GDF15 levels are directly detrimental to the brain [36].

In conclusion, our studies indicate that *Mic-1* KO mice display variations in a number of behavioural domains including anxiety, cognition and sensorimotor gating. Whilst there is some precedent for direct actions of MIC-1/GDF15 on the CNS, whether the observed effects are directly mediated or are indirect is not certain. To understand its modes of action, future research will have to examine the direct actions of MIC-1/GDF15 on brain regions including the hippocampus, amygdala and hypothalamus and also consider MIC-1/GDF15-induced changes to NPY, POMC, and IL-6.

## Supporting Information

S1 File Data Summary. All processed raw data of the various behavioural tests carried out are listed in the Excel file.
(XLSX)
